# Analysis of Chromatin Openness in Testicle Tissue of Yak and Cattle-Yak

**DOI:** 10.3390/ijms232415810

**Published:** 2022-12-13

**Authors:** Mengli Cao, Jie Pei, Lin Xiong, Shaoke Guo, Xingdong Wang, Yandong Kang, Xian Guo

**Affiliations:** 1 Key Laboratory of Yak Breeding Engineering of Gansu Province, Lanzhou Institute of Husbandry and Pharmaceutical Sciences, Chinese Academy of Agricultural Sciences, Lanzhou 730050, China; 2 Key Laboratory of Animal Genetics and Breeding on Tibetan Plateau, Ministry of Agriculture and Rural Affairs, Lanzhou 730050, China

**Keywords:** ATAC-seq, cattle-yak, yak, testis, spermatogenesis

## Abstract

Cattle-yak, a crossbreed of yak and cattle, which can exhibit obvious heterosis and can adapt to the harsh environmental conditions of the Qinghai Tibet Plateau (QTP). However, F1 cattle-yak were found to be sterile because they were unable to produce sperm, which adversely restricted the fixation of heterosis. Many prior attempts have been made to decipher the mechanism underlying the spermatogenesis stagnation of cattle-yak. However, the open chromatin region (OCR) map of yak and cattle-yak testes has not been generated yet. Here, we have analyzed the OCRs landscape of testicular tissues of cattle-yak and yaks by performing ATAC-seq technology. The OCRs of cattle-yak and yak testes displayed similar genome distribution and showed priority in intergenic regions, introns and promoters. The pathway enrichment analysis indicated that the differential OCRs-related genes were involved in spermatogenesis, involving the cell cycle, as well as Hippo, mTOR, MAPK, Notch, and Wnt signaling pathways. The integration of ATAC-seq and mRNA-seq indicated that the majority of the gene expression levels were positively correlated with chromatin openness. At the same time, we have identified a number of transcription factors (TFs) related to spermatogenesis and the differential expression of these TFs may contribute to the spermatogenesis stagnation of the cattle-yak. Overall, the findings of this study provide valuable information for advancing the research related to yak crossbreeding improvement and sperm production stagnation of cattle-yak.

## 1. Introduction

The yak (*Bos grunniens*) is a bovid animal, which possesses strong adaptability to harsh ecological environmental conditions such as extreme cold, hypoxia, and strong ultraviolet radiation. It is an indispensable omnipotent livestock found in the Qinghai-Tibet Plateau (QTP) and serves as an important means of production and living for the local herdsmen [1]. However, compared with ordinary cattle, the production performance of yak milk and meat is relatively low. Hence, to improve yak production performance, yaks have been usually crossed with cattle to take advantage of the various positive characteristics of these two species without damaging their resistance to harsh environmental conditions [2]. The offspring of cattle (♂) and yak (♀) crosses are called “true Pian Niu” or cattle-yak in English, whereas the offspring of yak (♂) and cattle (♀) are termed “false Pian Niu” [3]. In fact, both true and false Pian Niu display significantly improved meat and milk production performance [4]. Despite the obvious heterosis, the males of the F1 generation were found to be infertile due to their inability to produce the sperms. In addition, some studies have shown that multiple backcrosses can effectively dilute the sterility of cattle-yak, but with the increase of hybrid generations, cattle-yak can exhibit a substantial decline in adaptability and disappearance of heterosis [5]. However, due to the male sterility of the F1 generation cattle-yak, its heterosis cannot be fixed by cross-crossing, and the production performance of yak cannot be improved by crossing, which can markedly hinder the optimal development of the plateau yak industry.

Anatomical histology has shown that the circumference, diameter, and weight of cattle-yak testes were significantly smaller in comparison to those of yaks. The spermatogonia (SG) in the convoluted seminiferous tubule of the cattle-yak act as the main germ cells, and can produce only a few primary spermatocytes (PSC) and fewer secondary spermatocytes [6]. Cytogenetic analysis has indicated that cattle-yak has the same number of chromosomes (2n = 60) with that of cattle and yak [7], but the majority of PSC of cattle-yak was comprised primarily of a morphologically abnormal synaptonemal complex of autosomes and no XY bivalents were observed [8]. It has been speculated that arrest of the meiosis I could lead to infertility in male CY [9]. Spermatogenesis is a highly ordered and genetically coordinated process [3], that includes three distinct stages, mitotis of SG, meiosis of PSC to produce the haploid spermatids, and spermatogenesis in the seminiferous tubules [10]. The process is mainly regulated by the synchronized genes at the transcriptional, posttranscriptional and epigenetic levels [11]. However, with the development of sequencing technology, the mechanism of spermatogenic stagnation in cattle-yak has been extensively studied based on molecular genetics. Transcriptome analysis has revealed that undifferentiated SG marker genes and pro-apoptotic genes were upregulated in cattle-yak, whereas the different differentiation maintenance genes were downregulated, but the various genes related to the cell cycle progression in SG in mitosis and synaptonemal complex assembly in PSC meiosis related genes were hardly expressed in cattle-yak [12]. The whole-genome methylation level of the testis of cattle-yak was found to be significantly higher than that of yak, and the relative expression of the methyltransferase 3a (Dnmt3a) was also significantly higher [13]. Interestingly, it was found that PIWI/piRNA pathway genes (*PIWIL1*, *DDX4*, *PLD6*, *MAEL*, *FKBP6*, *TDRD1*, and *TDRD5*) were silenced due to methylation of the promoter region, which effectively disrupted the production of piRNAs in pachytene and interfered with spermatogenesis in cattle-yak [14]. However, the level of N6-methyladenosine (m6A) in the testis tissue of cattle-yak was substantially lower than that of yak [15]. bta-miR-34c can promote the transition of spermatogonia from mitosis to meiosis, and thus aid to maintain the stable number of spermatogenic cells in the seminiferous tubules, but the expression of bta-miR-34c is significantly downregulated in cattle-yak testis [4]. The target genes of lncRNA differentially expressed in cattle-yak and yak have been found to be mainly involved in the initiation of spermatogenic cell division as well as regulation of the cell cycle progression, homologous recombination, differentiation, and biological processes of apoptosis [16].

The transcriptome of cattle-yak testis was altered and epigenetic modification plays an important role. However, more research is needed to completely dissect the molecular mechanisms underlying spermatogenesis disorder in cattle-yak. Eukaryotic DNA is not bare, but is packaged into nucleosomes to form a beaded structure and further folded and packaged. Transcription of genes, however, requires unraveling this high-level structure, making DNA a bare state that allows various transcription machines to bind to it, forming open chromatin regions (OCRs). Importantly, the map of OCRs of the testes of yak and cattle-yak has not been generated, and different transcription factors (TFs) can bind to the DNA in OCRs in a sequence-specific manner and regulate the transcription process [17]. These OCRs are nuclease-sensitive due to the absence of nucleosomes. Assay for Transposase Accessible Chromatin with high-throughput sequencing (ATAC-seq) is a novel technique employed for assessing chromatin accessibility at the genomic level [18]. Based on the analysis of the male testicular cells by scATAC-seq, it was found that chromosomes 19 and 17 showed high chromatin accessibility, and specific motifs with high frequency at the different stages of spermatogenesis were detected, including *CTCF*, *BORIS*, *NFY*, *DMRT6*, *EN1*, *ISL1*, and *GLI3*. Moreover, combined with scRNA-seq data, it was found that TLE3 was specifically expressed in the differentiated sperm, PFN4 participated in the localization of actin cytoskeleton during meiosis, and the unique area upstream of *TLE3* and *PFN4* exhibited high accessibility [19]. ATAC-seq of the human adult spermatogonia stem cells (SSCs) displayed that open chromatin was significantly enriched in the binding sites of the various pioneer factors (NFYA, DMRT1, and hormone receptors) [20]. The findings of the above reports clearly indicated that the openness of chromatin plays an important role in regulating testicular development and spermatogenesis. Therefore, this study intends to use the ATAC-seq to detect the potential differences in OCRs in the testicular tissues of cattle-yak and yak on a genome-wide, and further analyze the possible effect of changes in OCRs on the gene expression level of cattle-yak by combining RNA-seq technology. Overall, the results can provide a reference for expanding the research related to male sterility of cattle-yak.

## 2. Results

### 2.1. Read Quality and Alignment

In this study, fresh ATAC-seq libraries of cattle-yak (*n* = 3) and yak (*n* = 3) testicular tissue preparations were sequenced on the Illumina Hiseq 2500 platform, and 150 bp paired-end reads were generated. A total of 78.26 G of data were sequenced from six different samples, raw reads with an average (±SD) per sample of 43,469,756 (±2,077,942) were initially collected. Thereafter by removing the linkers and low-quality reads, at least 40,851,986 clean reads per sample were obtained. Bowtie2 software was used to compare the clean read with the yak reference genome (version: LU_Bosgru_v3.0), and the comparison rate was about 94.2%. Then the bases were removed with a mass value of less than 30 and repetitive sequences introduced by PCR. Overall, finally, each sample has 19,748,788 (±1,636,271) valid reads (Supplementary Table S1).

**Figure 1:**
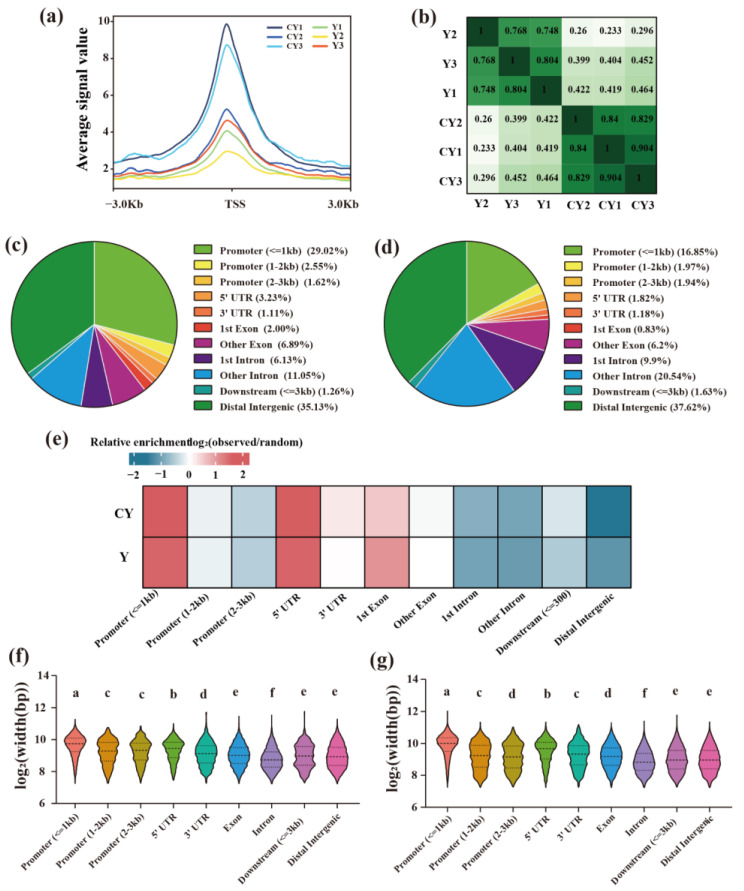
Open characteristics of the chromatin found in the testes of yaks and cattle-yak. (**a**) Distribution trend diagram of ATAC-seq signal near TSS, (**b**) Map showing the correlation analysis, (**c**) Yak and (**d**) cattle-yak peaks distribution diagram on the genomic functional elements, (**e**) Peaks relative enrichment diagram on the genomic functional elements, (**f**) Yak and (**g**) cattle-yak peaks width distribution violin diagram. CY: cattle-yak, Y: yak. The same letter in the violin figure indicates that the difference between the groups is not significant, and different letters indicate that the difference between the groups is significant (*p* < 0.05).

We assessed the library quality for each sample based on insert length as well as peak signal distribution, all the libraries displayed expected inserts, including abundant anucleosome-free and transnucleosome fragments (Supplementary Figure S1). The ATAC-seq signal was distributed differently around the transcription start site (TSS) for each sample, but the highest signal intensity was found at the TSS (Figure 1a), which suggested that the sequencing quality of each individual was relatively high. At the same time, the sample correlation test showed that the intra-group correlation coefficient was greater than the between-group correlation coefficient (Figure 1b), thus indicating that the intra-group sample repeatability was good.

### 2.2. Peaks Identification and Annotation

The peaks that existed in at least two distinct samples in the group were selected as peaks for analyses in this study. 55,974 and 130,211 peaks were identified in group yak andcattle-yak, respectively (Supplementary Tables S2 and S3). The distribution of peaks in group Y on the functional components of the genome showed that 35.13% of peaks were located in the distal Intergenic, 33.19% were promoters, and 17.18% were introns, 8.89% were located in exor, followed by 5’UTR (3.23%), downstream (≤300) (1.26%) and 3’UTR (1.11%) (Figure 1c). Distal intergenic (37.62%) was also preferred in the genomic distribution of cattle-yak peaks, followed by intron (30.44%), promoter (20.76%), exon (7.03%), 5’UTR (1.82%), Downstream (≤300) (1.63%) and 3’ UTR (1.18%) (Figure 1d). Regardless of the group yak or cattle-yak, more peaks were annotated to the distal intergenic, promoter, and intron. However, due to the different proportions of each functional region in the genome, we conducted relative enrichment analysis (observed/random value) on the functional region of the genome where the peaks were exactly located. The results indicated that the peak of the distal intergenic region and intron region was relatively low enriched, but highly enriched in the promoter (≤1 kb) and 5’ UTR (Figure 1e). According to the statistics of the width of the peaks detected in the different regions of the genome, it was found that the average width of peaks varies with the variation of the genome region, and there was inconsistency found between the cattle-yak group and yak group. However, the average width of peaks located in the promoter (≤1 kb) of the yak group or cattle-yak group was found to be significantly larger than that of other genomic regions (Figure 1f,g).

### 2.3. GO and KEGG Analysis of Differential OCRs-Related Genes

**Figure 2:**
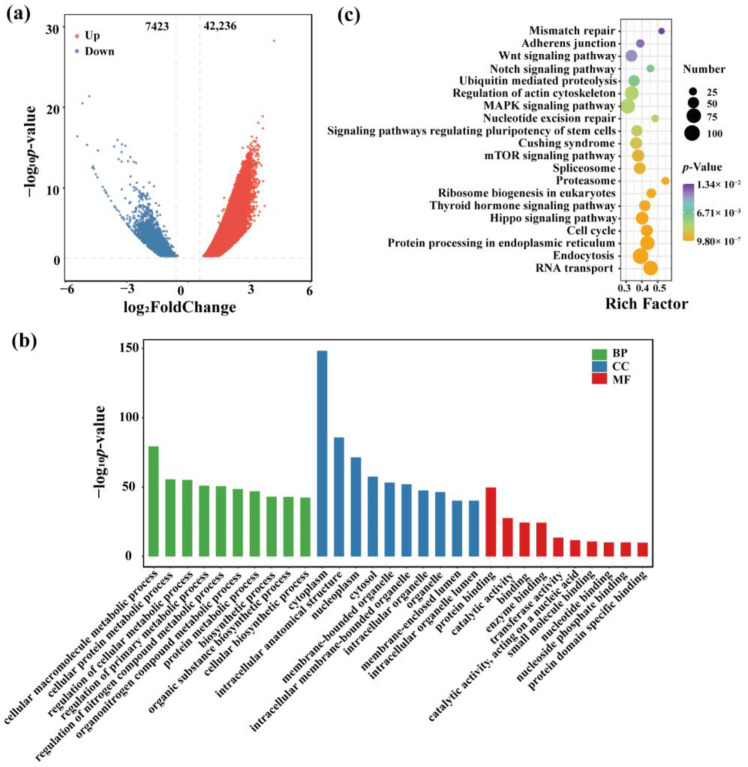
GO/KEGG analyses of the differential OCRs-related genes. (**a**) Volcanic map of the differential OCRs between cattle-yak and yak groups (| log_2_FoldChange | ≥ 0.58, *q* ≤ 0.05), (**b**) Annotation of the top 30 GO functions of the differential OCRs related genes, (**c**) The top 20 KEGG pathways of the differential OCRs related genes.

To investigate the role of OCRs in testicular development, we redefined the above-identified peaks. The difference of reading counts in the different peak groups was calculated, and DESeq2 was used to screen by threshold (| log_2_FoldChange | ≥ 0.58, *q* ≤ 0.05) to obtain the difference in OCRs. The results indicated that there were 49,659 different OCRs, among which 42,236 OCRs in the testicular tissue of the cattle-yak displayed increased openness and 7423 OCRs exhibited decreased openness (Supplementary Table S4 and Figure 2a). We paid more attention to the differential OCRs located in the promoter region with important regulatory functions in the genome, hence we next conducted Gene Ontology (GO) and Kyoto Encyclopedia of Genes and Genomes (KEGG) analysis on these OCRs-related genes. GO term analysis showed that these genes were mainly related to metabolism and binding: Biological processes: Cellular macromolecule metabolic process, cellular protein metabolic process, and regulation of cellular metabolic process; Cellular components: cytoplasm, intracellular anatomical structure, and nucleoplasm; Molecular functions: protein binding, catalytic activity and binding (Supplementary Table S5 and Figure 2b). KEGG enrichment analysis showed that these genes were closely related to testicular development and spermatogenesis, including of the cell cycle, Hippo, mTOR, MAPK, Notch, and Wnt signaling pathways (Supplementary Table S6 and Figure 2c). These results indicated that changes in the gene transcription level caused by significant changes in the openness of testicular OCRs might lead to poor testicular development and stagnant spermatogenesis in cattle-yak.

### 2.4. Combined Analysis of ATAC-seq and RNA-seq

**Figure 3:**
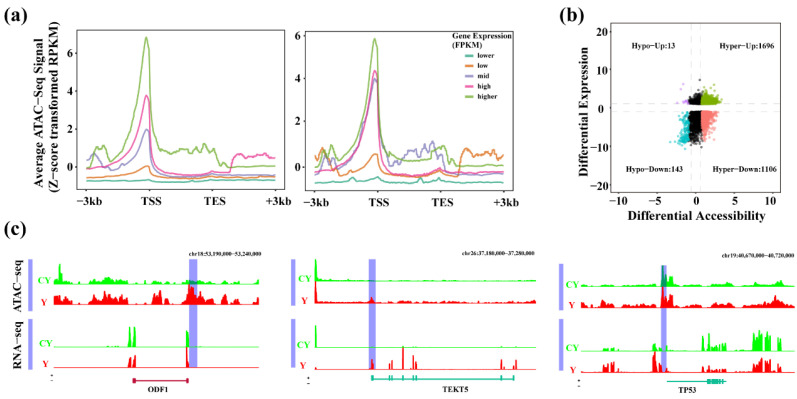
Correlation between ATAC signal and the gene expression in cattle-yak and yak groups. (**a**) ATAC signal and its potential relationship between upstream and downstream 3 kb and the gene expression, (**b**) Promoter region difference OCRs related genes and DEGs correlation analysis diagram, (**c**) Visual display of ATAC-seq and mRNA- seq signals in *ODF1*, *TEKT5* and *TP53* genes.

To explore the potential relationship between OCRs changes and gene expression levels in the testis of cattle-yak and yak, we integrated mRNA-seq data (accession number: GSE208693) of the samples in this study for further analysis. According to the FPKM value of the gene expression, we divided the genes into five distinct groups (lower, low, mid, high, and higher), and calculated the chromatin openness level at the different gene positions. The results showed that the expression levels of most genes were positively correlated with the level of chromatin openness, that is, the higher the gene expression level, the stronger the chromatin openness. In addition, we found that except for the lower group, the chromatin openness level of the other four groups exhibited the highest peak near the TSS (Figure 3a). We paid more attention to the promoter region of OCRs, so the correlation between DEGs (| log_2_FoldChange | ≥ 1, *q* ≤ 0.05) and promoter region difference OCRs (| log_2_FoldChange | ≥ 0.58, *q* ≤ 0.05) was analyzed. The chromatin open level and mRNA level of 2958 genes were found to be significantly different, among which 1696 genes upregulated chromatin open level and mRNA expression level, 1106 genes increased chromatin open level but downregulated mRNA expression level, 13 genes decreased chromatin open level but upregulated mRNA expression level, whereas open chromatin level and mRNA level expression of 143 genes were significantly down-regulated (Supplementary Table S7 and Figure 3b). To analyze that how chromatin openness can potentially regulate gene expression, we analyzed the expression of spermatogenesis-related genes *ODF1* (outer dense fiber of sperm tails 1) and *TEKT5* (tektin 5), as well as the cell cycle-related gene *TP53* (tumor protein p53) ATAC-seq and RNA-seq signals. The results indicated that the level of ATAC-seq signal in the promoter region of the related genes was significantly higher in comparison to that found in other locations (Figure 3c).

### 2.5. Motif Analysis of Differential OCRs

**Figure 4:**
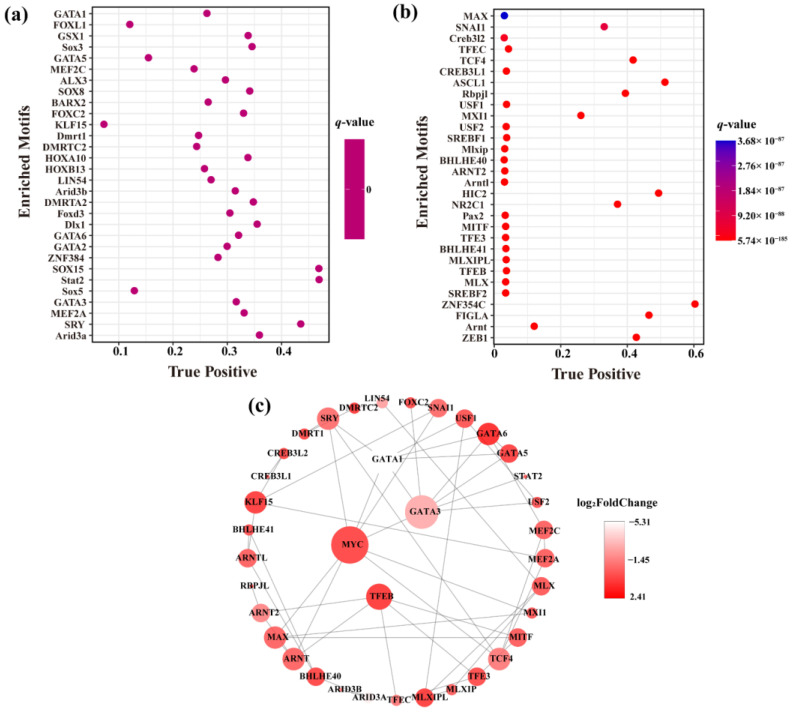
Motif analysis of the differential OCRs. The openness of cattle-yak OCRs can increase (**a**) and decrease (**b**) Motif enrichment results, and (**c**) The interaction network between the different TFs. The size of a node indicates interaction. The color of nodes indicates the differential expression level of TF (log_2_FoldChange).

It has been established that TFs and other regulatory proteins are required to identify the specific motifs to combine with DNA to initiate gene expression. We used the MEME Suite tool to perform Motif scanning and enrichment analysis on the differential OCRs in the JASPAR database (Supplementary Tables S8 and S9). Figure 4a,b shows the Motif enrichment of top30 with increased and decreased OCRs in the testis of cattle-yak, respectively. We have subsequently constructed the network regulation map of TFs with top 30 OCRs increase and decrease in the testis of cattle-yak (Supplementary Table S10 and Figure 4c). We found that *MYC* (myelocytomatosis oncogene), *GATA3* (GATA binding protein 3), *GATA1,* and *TFEB* (transcription factor EB) were the most connected TFs, and the expression levels of these TFs were observed to be significantly different between cattle-yak and yak groups. They can cooperate closely with several other TFs in the regulatory network and might play an important role in regulating the optimal development of the cattle-yak testis and spermatogenesis.

## 3. Discussion

The openness of cis-regulatory element promoters and enhancers to DNA-binding proteins is substantially limited by the local structure of chromatin. It has been found that only OCRs without nucleosome encapsulation can bind to TFs and recruit RNA polymerase into the core promoter to initiate transcription [21]. In addition, compared with MNase-seq and DNase-seq, ATAC-seq possesses the advantages of high efficiency as well as low cell input requirements, and facilitates the simultaneous identification of open chromatin regions, nucleosome localization, and regulatory motifs. The greatest innovation of ATAC-seq is the potential application of Tn5 transposase. Although Tn5 transposase can produce slight bias due to the sequence-dependent binding, this transposition bias can be corrected by developing innovative computing tools [22]. Therefore, this study systematically analyzed the openness of chromatin in cattle-yak and yak testis by using ATAC-seq and mRNA-seq techniques. Of course, the interaction between protein and DNA is often highly dynamic, and we are unable to reveal some specific interactions, but in this study, we have obtained relatively stable OCRs.

The peaks in both cattle-yak and yak groups exhibited a higher proportion distribution in the distal intergenic and intron, followed by the promoter, then exor, 5’UTR, downstream, and 3’UTR. This might be probably caused by the high proportion of the distal Intergenic and intron in the yak genome [23]. This result was consistent with the results of other studies on yak OCRs [24]. The relative enrichment analysis of the peaks in the functional region of the genome showed that the promoter region was significantly enriched, and the ATAC-seq signal intensity of all the samples was the highest at the TSS, which indicated that most of the cis-regulatory elements in the genome are close to the core promoter region. This was consistent with the study of OCRs by Fan et al. [25] during embryogenesis in five different species. We also found that the average width of peaks in the promoter (≤1 kb) of either yak or cattle-yak was significantly greater than that of other regions of the genome. Yue et al. [26] found in the study about OCRs in the skeletal muscle during pig embryonic development that when the proximal promoter region of the gene contained multiple or longer OCRs, it often exhibits a higher expression level, which indicated that the change of chromatin openness in the promoter region can affect the expression level of the various related genes.

The functional enrichment analysis of the differential OCRs-related genes in the promoter region showed that differential OCRs-related genes can be significantly enriched in the pathways related to spermatogenesis, such as cell cycle, mTOR, Notch, and Wnt signaling pathways. The notch signaling pathway is essential for initiating mitotic arrest and enables the maintenance of male germ cells’ identities. A prior study has reported that the conditional activation of the Notch1 intracellular domain in the germ cells could lead to an increase of germ cell apoptosis, reduction in the sperm counts, and progressive loss of testis weight with age [27]. Another study showed that Notch 1–3 as well as ligands Dll-1 and Dll-4 were expressed in Leydig cells, and Notch signaling suppressed the proliferation of Leydig cells and induced G0/G1 arrest [28]. These studies indicated that the Notch signaling pathway plays an important role in the process of spermatogenesis, and pharmacological inhibition of the Notch signaling pathway can lead to spermatogenesis defects [29]. mTOR is considered to be the central aggregate in the PI3K/AKT/mTOR signaling pathway, which is actively involved in the regulation of cellular metabolism, growth, and proliferation. It should be noted that mTOR plays an important role in maintaining and differentiating spermatogonial stem cells and regulating the redox balance as well as the metabolic activity of Sertoli cells [30], where Sertoli cells play an important role in providing nutrition and support during spermatogenesis. It was found in this study that *TGFB2* (transforming growth factor-β2) and *TGFB3* were significantly enriched in the cell cycle, Hippo, MAPK, and other signaling pathways related to spermatogenesis. They are two of the three subtypes of transforming growth factor-β that have been identified in mammals, and play an important role in regulating the growth, differentiation, and development of the various cells in living things. Transforming growth factor-β can act on the retinoblastoma protein and cyclin-dependent kinases to block the late G1 phase of the mitotic cell cycle and thus inhibit the cell growth [31], as well as might hinder the normal mitosis of cattle-yak spermatogonial stem cells. The above results indicated that differential OCRs-related genes play an important role both in testicular development and spermatogenesis.

Through the joint analysis of ATAC-seq and mRNA-seq data, we further explored the possible interaction between chromatin openness and gene expression levels. We found that the expression level of most genes was positively correlated with the chromatin opening level. Moreover, the genes exhibited the highest level of ATAC-seq peak near TSS, which was consistent with the report in the study of early bovine embryos [32] and yak adipocytes [24]. This finding also conforms to the general view that transcription factors regulate gene expression mainly by activating the expression of specific genes. However, there are some inconsistent signals related to the variable trend of ATAC-seq and mRNA-seq, which could be possibly due to the influence of DNA methylation or other epigenetic modifications. Alternatively, it could be attributed to the existence of some inhibitory TFs, for example, *CTCF* binding sites are mainly concentrated in intergenic regions as well as introns and overlap with enhancer and promoter sequences. It has been reported that the binding of *CTCF* with promoter or enhancer regions usually plays an inhibitory role [33]. Moreover, when the *SP3* factor and active *SP1* factor bind to the same site, they can act as passive repressors by preventing the active factors from binding to DNA binding sites, thus preventing activation [34]. These factors can inhibit the gene transcription directly by binding with the basic transcription complex or indirectly by inhibiting the positive factors, which ultimately leads to the failure of the initiation of the gene transcription. This may be the primary reason for the inconsistent signals observed between ATAC-seq and mRNA-seq.

In this study, we conducted an enrichment analysis on the motif of differential OCRs. The TFs identified with more connections were *MYC*, *TFEB*, *GATA1,* and *GATA3*. MYC is a member of the helix-loop-helix leucine zipper superfamily, including *C-myc*, *N-myc*, and *L-myc*. *C-myc* is an androgen-regulatory gene expressed in Sertoli cells. A number of studies have shown that in the transgenic rats expressing *C-myc*, spermatogenesis at the primary spermatocyte level was blocked, and apoptotic DNA fragments increased significantly [35]. *TFEB* is a major regulator of lysosomal biogenesis, autophagy, and endocytosis. The regional expression and activation of TFEB were closely related to the retinoic acid signal, which can effectively promote cell migration in the blood-testis barrier and transport along the seminiferous epithelium [36]. The *GATA* family is a kind of transcription regulator with a zinc finger structure, which plays an important role in regulating organ morphogenesis, cell proliferation, and gender differentiation. *GATA1* and *GATA3* are developmental stage and spermatogenic cycle-specific regulators of gene expression in the Sertoli cells [37,38]. A prior study has indicated that *GATA1* occupied the *MYC* promoter in vivo, thus suggesting the direct mechanism of gene inhibition. Surprisingly, the forced expression of *MYC* prevented the cell cycle arrest induced by *GATA1* [39]. Another study showed that *C-myc* is a key target for *GATA3* and can promote cell proliferation [40]. In our study, the core TFs *MYC* interacted with *GATA1* and *GATA3*, which may contribute to the spermatogenesis defect of the cattle-yak, but the specific mode and mechanism of its role in spermatogenesis need further analysis.

## 4. Materials and Methods

### 4.1. Ethics Statement

All experimental procedures involved in this study were reviewed and confirmed by the Animal Administration and Ethics Committee of Lanzhou Institute of Husbandry and Pharmaceutical Sciences, Chinese Academy of Agricultural Sciences (SYXK-2014-0002).

### 4.2. Animals and Sample Collection

Three male Gannan yaks (Y1, Y2, and Y3) were obtained from Linxia County, Linxia Hui Autonomous Prefecture. Three male cattle-yaks (CY1, CY2, CY3) were the hybrid offspring of Jersey cattle (♂) and Gannan yak (♀), obtained in Xiahe County, Gannan Tibetan Autonomous Prefecture. All animals were 4 years old. After the yak was euthanized, the testicular tissue was collected, and the testicular tissue of the cattle-yak was collected by veterinary surgery. The samples were sent to the Lanzhou Institute of Husbandry and Pharmaceutical Sciences, Chinese Academy of Agricultural Sciences, for further experiments.

### 4.3. ATAC-seq Library Preparation, Sequencing, and Analysis

ATAC-seq was performed as reported previously [18,41]. Briefly, nuclei were extracted from samples, and the nuclei pellet was resuspended in the Tn5 transposase reaction mix. The transposition reaction was incubated at 37 °C for 30 min. Equimolar adapter 1 and adapter 2 were added after transposition, and PCR was then performed to amplify the library. After the PCR reaction, the libraries were purified with the AMPure beads and their quality was assessed with Qubit (Thermo Scientific^TM^, Waltham, MA, USA). The clustering of the index-coded samples was performed on a cBot Cluster Generation System using TruSeq PE Cluster Kit v3-cBot-HS (Illumina^TM^, San Diego, CA, USA) according to the manufacturer’s instructions. After the cluster generation, the library preparations were sequenced on an Illumina Hiseq 2500 platform and 150 bp paired-end reads were generated.

The raw data was first used in Trimmomatic [42] to remove the sequencing adapters and low-quality bases with the parameters ILLUMINALIP:NexteraPE-PE.fa:2:30:10:8:true MINLEN:8. After the trimming, the quality of the sequencing data was assessed by using FastQC. Clean Data was aligned with the yak reference genome (LU_Bosgru_v3.0) sequence using Bowtie2 [43] software parameter-X 2000. Then, Picard was used to mark the repeats to determine the accurate number of the repeat reads and Samtools (v1.9) [44] to remove different fragments that were not aligned with the reference sequence as well as reads with the base quality value less than 30 and repeat sequences introduced by PCR. Peak detection for each sample was performed by MACS3 [45] with parameters-f BED–nomodel–shift-75–extsize 150-B–SPMR–keep-dup = all, and the data reproducibility assessment was carried out on the detected peaks. The peak signals were visualized with Deeptools (Version 3.5.0, University of Oxford, Oxford, UK) [46].

The peaks co-existing in at least 2 samples within a group were annotated by employing ChIPseeker [47]. Thereafter, by using the BEDtools shuffle tool (Version 2.30.0, Kunlan Laboratory, Salt Lake City, UT, USA), with the above peaks file as a reference, random regions were extracted from the chromosome length file, and ChIPseeker was sued to annotate these random regions, and the number of these random regions located in each element of the genome were counted. The above process was repeated 5000 times, and the average value of the annotation results of random regions in the different elements of the genome was a random value. The number of different peaks detected in this study is an observation value. The relative enrichment of peaks in the different regions of the genome was plotted in R. GraphPad Prism8 was used to draw a violin plot of the widths of peaks in the different regions of the genome, and SAS9.4 was used to perform an analysis of variance on the widths of peaks in the different regions of the genome.

R package DiffBind was used to analyze the differential OCRs. First, an overlap of each sample peak was taken to obtain the summit and expanded 200 bp upstream and downstream with the summit as the center, to redefine the peak, and calculate the reads counts of each sample on the overlapping peak. The reads counts on the peaks of each sample were then normalized for the correction, by using the difference comparison tool DESeq2 [48], and the filter threshold was set to: | log_2_FoldChange | ≥ 0.58, *p* ≤ 0.05. We used the R package clusterProfiler4.0 [49] to perform Gene Ontology (GO) and Kyoto Encyclopedia of Genes and Genomes (KEGG) enrichment analysis of the differential OCRs-related genes (genes represented by the TSS closest to the COR center) in the promoter region. Both motif scanning and enrichment analysis of the differential OCRs were performed in the JASPAR database (http://jaspar.genereg.net, accessed on 12 September 2021) by using MEME Suite [50].

### 4.4. RNA-seq Library Preparation, Sequencing, and Data Processing

Total RNA from samples was extracted using TRIzol reagent. The NanoDrop 2000 Spectrophotometer (Thermo Scientific^TM^, Waltham, MA, USA) was employed for assessing the RNA’s quantification and purity. Agilent 2100 Bioanalyzer (Agilent Technologies^TM^, Santa Clara, CA, USA) was used for probing RNA integrity. Finally, the library was created as per the manufacturer’s manual by employing the TruSeq Stranded mRNA LT Sample Prep (Illumina^TM^, San Diego, CA, USA). The sequencing was then performed by using an Illumina HiSeq 2500, generating 150 bp paired-end reads. All the sample raw data used Trimomatic [42] to trim the various adapters and low-quality bases. The clean data was then mapped onto the genome (version: LU_Bosgru_v3.0) using HISAT2 [51]. Finally, Fragments Per Kilobase of exon model per million mapped fragments (FPKM) for individual genes was calculated by using Cufflinks, and read counts for each gene were obtained by HTSeq-count [52]. DESeq2 [53] in the R package was employed to perform the differential expression analysis depending upon | log_2_FoldChange | ≥ 1 and *q* ≤ 0.05.

### 4.5. Integration of ATAC-seq and RNA-seq

We next divided all the genes into five different groups (lower, low, mid, high, and higher) according to the quintile threshold of the FPKM value of the gene expression. R/Gviz was used to study the distribution trend of the ATAC-seq signal in the gene body and its upstream and downstream 3 kb regions at the different expression levels. IGV browser was used to display the visualized results at the whole genomic level. STRING database was employed to generate TF tuning network and visualize it in Cytoscape (Version 3.7.1, Paul Shannon, MA, USA).

## 5. Conclusions

In summary, our study described the landscape of OCRs in the testes of both yak and cattle-yak. The integration of ATAC-seq and mRNA-seq indicated that most gene expression levels were positively correlated with chromatin openness. Additionally, we have identified several TFs related to spermatogenesis, and the differential expression of these TFs might contribute to the spermatogenesis stagnation of the cattle-yak. The findings of this study provide valuable information related to the research about yak crossbreeding improvement and sperm production stagnation of cattle-yak.

## Data Availability

Raw reads of ATAC-seq of yak and cattle-yak testis are available at SRA (accession number: PRJNA894119).

## Supplementary Materials

The following supporting information can be downloaded at: https://www.mdpi.com/article/10.3390/ijms232415810/s1.
