# Supplementary figures and images for: Analysis of Chromatin Openness in Testicle Tissue of Yak and Cattle-Yak
Source: Int J Mol Sci. 2022 Dec 13;23(24):15810. doi: 10.3390/ijms232415810 (PMC9785434; doi:10.3390/ijms232415810)

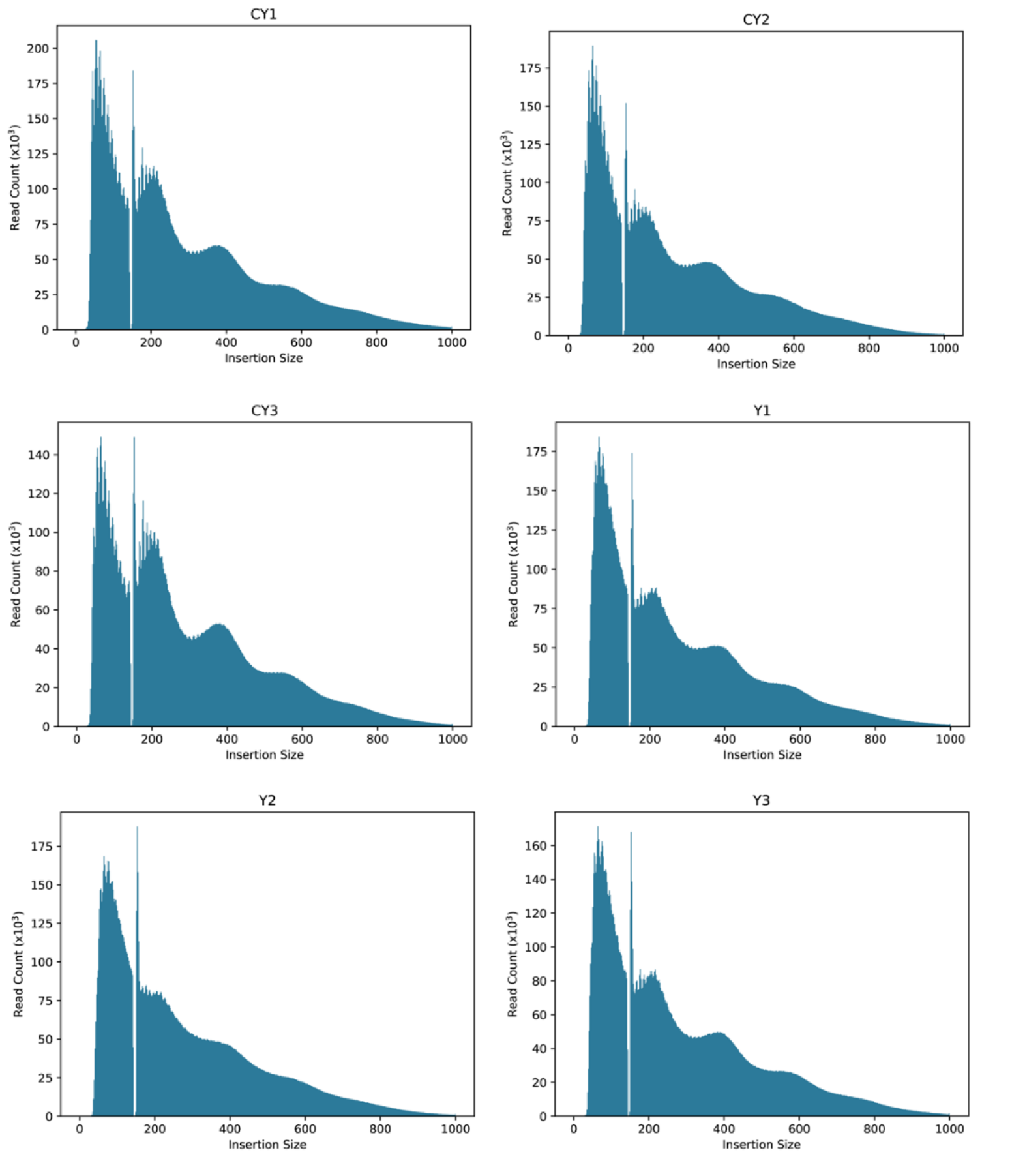

Supplement: Supplementary file 1 [file ijms-23-15810-s001.zip › Supplementary Figure S1.tif]
